# Assessing Effects of Diet Alteration on Carbohydrate–Lipid Metabolism of Antipsychotic-Treated Schizophrenia Patients in Interventional Study

**DOI:** 10.3390/nu15081871

**Published:** 2023-04-13

**Authors:** Mariola Friedrich, Joanna Fugiel, Joanna Sadowska

**Affiliations:** 1 Department of Applied Microbiology and Human Nutrition Physiology, Faculty of Food Sciences and Fisheries, West Pomeranian University of Technology, ul. Papieża Pawła VI 3, 71-459 Szczecin, Poland; 2 Social Welfare Home Names Dr. E. Wojtyły in Szczecin, ul. Stanisława Herakliusza Lubomirskiego 7, 71-505 Szczecin, Poland

**Keywords:** schizophrenia, carbohydrate–lipid metabolism, diet correction, health-promoting nutrition-related education

## Abstract

This study aimed at finding whether healthy eating habits could be introduced to and maintained by chronically mentally ill permanent residents of a nursing home. Of interest was also if the effects of the dietary intervention would be observable as improved carbohydrate and lipid metabolism indicators were selected. Assays covered 30 antipsychotics-treated residents diagnosed with schizophrenia. The prospective method applied involved questionnaires, nutrition-related interviews, anthropometric measurements, and determination of selected biochemical parameters of the blood. The dietary intervention as well as the parallel health-promoting nutrition-related education was aimed at balancing the energy and nutrient contents. Schizophrenia patients were shown to be capable of accepting and observing the principles of appropriate nutrition. The intervention was strong enough to result in a significant blood glucose concentration drop to the reference level in all patients, regardless of the antipsychotic they were treated with. The blood lipid levels also improved, but the reduction in triacylglycerols, total cholesterol and LDL-cholesterol levels was significant in the male patients only. Nutritional changes were reflected in overweight and obese women only, in body weight reduction and in waist adipose tissue loss.

Introduction

An increase in the incidence of mental illnesses, including schizophrenia, has been observed in recent years. Globally prevalent cases of schizophrenia rose from 13.1 million in 1990 to 20.9 million cases in 2016 [1]. In 2019, schizophrenia affected approximately 24 million people (0.32%) worldwide [2]. In the European Union in 2018, 1.5 million people suffered from schizophrenia (0.3%) [3]. There is also an increase in the incidence of metabolic diseases associated with carbohydrate–lipid metabolism disorders. In the decade from 2003 to 2013, the prevalence of metabolic syndrome in Polish adults increased by 3.3 percentage points in women (26.6% vs. 29.9%) and by 8.8 percentage points in men (30.7% vs. 39.4%) [4]. 

As shown by numerous studies, carbohydrate–lipid metabolism disorders are more frequent in schizophrenics and bipolar patients than in the population as a whole. The reasons for this are not entirely clear, but they are likely to be multifactorial. The increased risk of metabolic complications may result from a direct effect of mental illness or an indirect effect of its treatment on the metabolism, hormonal status or behavioral patterns of patients that increase the risk of obesity. Common risk factors for mental illness and metabolic disorders also include genetic predisposition, sleep disorders and stress [5,6]. There is also evidence of a link between an unhealthy diet that promotes obesity and mood disorders, including anxiety and depression [7,8]. The reasons are sought in effects of more frequent insulin resistance in those patients [9]. Drugs applied to treat schizophrenia and bipolarity are an additional factor enhancing or generating metabolic disorders [10,11] and contribute to, inter alia, insulin resistance [12] and its consequences manifested as body weight gain, type 2 diabetes, hyperlipidemia, etc. [13,14,15].

The results of the research of Kalinowska et al. [16], Zomer et al. [17], Gilbody et al. [18], Ward et al. [19] and Tylec et al. [20] prove the validity, possibility and effectiveness of various lifestyle interventions in improving the quality of life in people with schizophrenia. Alleviating the symptoms of schizophrenia, in addition to pharmacological treatment, may also be supported by dietary components, including curcumin, stigmasterol, vitamins, exogenous melatonin and cannabidiol [21]. The Literature data also indicate that it may be beneficial to use caloric restrictions and inter alia docosahexaenoic acid, resveratrol, isoflavones and anthocyanins, which limit damage and support repair processes in DNA [22].

Therefore, it has been decided to find if, and to what extent, it is possible to apply health-promoting nutrition habits that would beneficially influence the system of antipsychotic-treated schizophrenia patients staying in a 24-hour social nursing home, and whether the effects would be demonstrated in selected carbohydrate–lipid metabolism indicators.

The presented research results are part of a large project, the results of which were published in the work of Friedrich et al. [23], in which the results and their discussion for the entire group of residents (*n* = 52) staying in the nursing home, divided into schizophrenic patients and other patients, are presented. This division drew our attention to the different metabolic responses of schizophrenia patients to the applied correction of nutrition. Therefore, this study presents and discusses the results of only the group of patients with schizophrenia (*n* = 30), further eliminating from the group of women three sisters whose metabolic reactions to the change in nutrition were different from the other patients with schizophrenia and could result from genetic conditions.

## 2. Materials and Methods

### 2.1. Participants

The study, which was carried out for 3 years, is part of the interventional research based on examining a total of 52 patients: 18 females aged 45–80 (64 ± 10.2) and 34 males aged 27–80 (59.2 ± 12.5), all staying in a 24-hour nursing home (NH) for the chronically mentally ill, for at least 4 years. In this work, effects of dietary changes were assessed from anthropometric indicators and those related to the carbohydrate–lipid metabolism of 30 patients diagnosed for schizophrenia, treated with antipsychotics. The group consisted of 12 females aged 45–80 and 18 males aged 52–80, staying in NH from 4 to 6 years, treated with, inter alia, clozapine (12 individuals: 8 females and 4 males); olanzapine (15 patients: 5 women and 10 men); haloperidol (3 patients: 1 female, 2 males); risperidone (3 patients: all female); and quetiapine (2 patients, both male). Some patients were treated with 2 different antipsychotics. The antipsychotics used are known to affect carbohydrate–lipid metabolism. 

The study and the associated diet modifications were approved by the NH director; 21 non-incapacitated patients gave their consent in writing, as did 9 legal counsels of the totally incapacitated ones. The project was approved by the Regional Chamber of Physicians’ Bioethical Commission (no. 14/KB/V/2013). The kitchen staff also expressed their acceptance for the introduced changes, especially in the technology of meal preparation.

A prospective method with questionnaires, diet assessment interviews, anthropometric measurements, and determination of selected biochemical blood parameters was applied. Primary care physicians, therapists, and medical records were used as sources of information on the patients’ health condition (both current illnesses and health history), mental disorders diagnosed, pharmaceuticals, wellbeing, destructive behaviors, etc. 

### 2.2. Diet and Nutrition

Prior to commencing the actual study, each patient was interviewed (using the questionnaire developed by the authors) to identify their most liked and most disliked foods and dishes. The information thus provided was used to modify the diets and to develop new menus. 

Once nutrition supervision was introduced to monitor patients’ nutrition and eating habits throughout the project duration, the diet’s energetic and nutritional qualities were balanced, with due consideration to each patient’s gender and age, and particular attention was paid to the major disease and accompanying disorders. Care was taken to change the sources of the basic nutrients (proteins, carbohydrates, and lipids) to those more frequently recommended or were health-promoting. Furthermore, the diets were supplemented with higher amounts of probiotic yogurts, natural fermented products, vegetables and fruits, natural herbs and spice mixes as well as higher amounts of liquids (water). The diets were altered by eliminating milk, highly processed, refined and preserved products, and caffeine-containing beverages, and the amount of salt and sucrose added to dishes was limited. The meals were rendered more diverse, and some technologies of meal preparation were changed (e.g., cooking and frying of meats were replaced by stewing and roasting). When developing decadal menus, a morning snack (“the second breakfast”) was added for all the patients, as was an afternoon snack and cooked supper; fruits and vegetables replaced sandwiches as the between-meals snacks. After consultation with the physician, from October to March, supplementation with fish oil containing 3.6 µg of vitamin D3 in a daily dose was also introduced.

The changes—fully accepted by the patients—were introduced at a slow pace over a longer period. 

In addition to occupational therapy, the patients received health-promoting nutrition education in the form of lectures (adjusted to the patients’ and their legal representatives’ perception and comprehension abilities), presentations as well as workshops focused on culinary therapy and nutrition. The efficacy of those activities was assessed during special meetings via role-playing, reviews, choice tests and nutrition-focused contests.

In this work, effects of changes in the dietary regime were assessed by comparing 30-day periods, in autumn/winter, before the changes were introduced, and a year later, when the regime alteration had been completed. The comparison included the amount and type of additional foods eaten by the patients, as reported by the non-incapacitated patients. Information from totally or partly incapacitated patients was collected from their first-contact caregivers (members of therapeutic teams and the NH staff). The total amount of food consumed by each patient was reduced by the amount of food left (and weighed) on plates after each meal and was brought back to the kitchen. 

The energy and nutritional value of the daily food ration was calculated using the “*Dieta 5.0*” program of the Institute of Nutrition and Food, taking into account the losses occurring during the preparation of meals. It was assumed that the energy provided by basic nutrients (proteins, fats, carbohydrates, including sucrose) should constitute 15, 55, 30 and <10% of the total energy value of the diet, respectively. Dietary fiber and cholesterol intakes were compared to levels recommended for the prevention of obesity and other non-contagious diseases (>25 g for fiber and <300 mg for cholesterol). The energy value of the diet was compared with the estimated energy requirement (EER); intake of vitamins and minerals was compared with the applicable standards at the level of estimated average requirements (EAR) for folates, B_1_, B_2_, B_6_, D_3_, C, Ca, Mg, Fe, Zn, Cu or the adequate intake (AI) for vitamins D_3_ and E as well as for those of K, Na and water [24]. All calculations were made for food intake for 30 days before and 30 days after diet adjustment.

For individual meals and daily food rations, the glycemic load was calculated [25], taking into account the values contained in the international tables of glycemic index and glycemic load [26] and in The University of Sidney database [27].

During adjustment of the diet, the financial possibilities of the institution, the relevant provisions of the Regulation of the Minister of Labour and Social Policies on nursing homes [28], nutritional standards, seasonality of food products and taste preferences of patients were taken into account. 

### 2.3. Anthropometric Analyses

Anthropometric measurements were taken using the classical Martin technique [29], in the morning, in the NH nurses’ office. Body height was measured with a SECA 215 stadiometer to 0.1 cm; waist and hip circumference was measured using a Gulick anthropometric tape to 1 mm; body weight was determined using a RADWAG WPT-200.0 medical scale to 0.1 kg. The measurements were taken in 3 replicates. Body Mass Index (BMI) was calculated from the formula: body weight (kg)/squared height (m^2^) and was compared to reference values of the World Health Organization (WHO) classification for patients aged 18-64 and of Lipschitz [30] for older than 65, respectively. Waist circumference (WC) and hip circumference (HC) were measured, waist-to-hip ratio (WHR) was calculated from the formula WHR = waist circumference (cm)/hip circumference (cm) and waist-to-height ratio (WHtR) from the formula WHtR = waist circumference (cm)/height (cm) [31]. 

### 2.4. Biochemical Assays

Blood analyses were performed before the modification of the diet and one year after its introduction. Blood was drawn from the basilic vein between 7:30 and 8:00 a.m. before breakfast by NH nurses who knew and were familiar with the patients (ensuring their consent and cooperation). The analyses were performed in the Laboratory of the Central District Public Hospital in Szczecin. Plasma concentrations of glucose (GL), triacylglycerols (TG), total cholesterol (TC) and LDL and HDL cholesterol fractions were determined by the enzymatic colorimetric method on the COBAS C6000 apparatus, using Roche Diagnostics reagents. The recommendations of Neumeister et al. [32] were taken into account when interpreting the results for glucose and the European Heart Journal [33] for lipids.

### 2.5. Statistical Treatment

Data on the patients’ diet energy and nutritional levels as well as on the blood parameters analyzed, collected before and after diet adjustment, were—following testing for distribution normality (the Shapiro–Wilk test) and homogeneity of variance (Levene’s test)—tested for significance, using Student’s t test for paired samples (*p* ≤ 0.05 and *p* ≤ 0.01); the analyses were run using the Statistica^®^ 12.0 software (TIBCO, Palo Alto, CA USA). 

## 3. Results

Nutritional habits, separately in females and males, were assessed prior to and following diet alteration, by analyzing, each time, 360 menus of women (a total of 720) and 540 menus of men (a total of 1080). Before diet modification, the mean energy content of daily food ration (including snacking and meal remains) in both male and female patients was higher than the recommended standard. It involved a too high consumption of animal protein, fats, cholesterol, and carbohydrates. After the diets were modified, the mean diet energy content was found to be significantly reduced, almost to the recommended level in male patients. In addition, the diet showed decreased contributions of animal protein, fats, cholesterol, and total carbohydrates; the consumption of dietary fiber and liquids (water) increased (Table 1). 

The proportions of major nutrients and the amount of energy they contributed (Table 2) showed significant changes occurring following the dietary intervention. 

Changes in the diet also translated into statistically significant changes in the content of vitamins and minerals in the daily food rations. From the point of view of the biosynthesis of neurotransmitters and the functioning of the nervous system, they are presented in Table 3. 

Changes in the diet were also reflected in a statistically significant reduction in the value of the 24-hour diet glycemic load, despite the introduction of an additional meal, which was an afternoon snack (Table 4). Additional eating had the largest share in this respect.

In the female schizophrenia patients, diet alteration translated into a significant reduction in body weight and BMI as well as a reduction in HC and WC, the last being, however, statistically non-significant (Table 5). On the other hand, the male patients showed no significant changes in any of those metrics, although favorable changes were observed in some of those patients. 

Analysis of effects produced by changed dietary habits on the selected carbohydrate–lipid metabolism parameters in the antipsychotic-treated schizophrenia patients showed, in females, a significant reduction in the glucose concentration. Glucose reduction was accompanied by a decline in triacylglycerol, total cholesterol, and LDL cholesterol concentration. These effects were, however, non-significant, as was a small increase in the HDL cholesterol concentration and HDL-C/TC ratio (Table 6). 

On the other hand, the beneficial changes observed in male patients, including a reduction in the concentrations of glucose, triacylglycerols, total cholesterol, and LDL cholesterol, proved significant. The HDL cholesterol after diet modification was similar to that observed before the dietary changes (Table 6).

## 4. Discussion

Carbohydrate–lipid metabolism disorders, with their consequences as type 2 diabetes, dyslipidemia, and coronary diseases, are twice as frequent in schizophrenia patients compared to the general population [34]. Recent research provides grounds to presume that schizophrenia and coronary diseases share genetic underpinnings [35,36]. In addition, as shown by numerous studies, some drugs used in schizophrenia treatment enhance carbohydrate–lipid metabolism disorders and type 2 diabetes. This is particularly relevant with respect to clozapine and olanzapine [37]. For example, clozapine and risperidone have been suggested to inhibit glucose transport to cells [38] and glucose metabolism [39], thus contributing to insulin resistance and its consequences.

In this context, the significant reduction in the glucose concentration in all 12 female patients and in 18 males (as shown by individual analysis), which followed diet modification, was particularly interesting. It demonstrated that dietary recommendations were adhered to in the patients and were beneficial in all of them, regardless of the antipsychotic used.

The drop in glucose concentration was produced by, inter alia, a lower consumption of simple sugars and processed complex carbohydrates, which are rapidly digested and release glucose in higher amounts. The increased uptake of complex carbohydrates from whole-grain cereals, brown rice, legumes, and vegetables contributed to glucose level reduction as well. The changes introduced in the nutrient sources resulted in a decrease in the proportion of sucrose in the diet and a decrease in GL (Table 2 and Table 4). A physiological mechanism controlling glucose removal from the blood was taken advantage of here; the efficacy of this mechanism is positively correlated with the type, source, and amount of dietary carbohydrates and the associated glucose tolerance factor (GTF) [40]. The extended digestion of such carbohydrates and the slow absorption of the glucose released were accompanied by increased consumption of various dietary fiber fractions in antipsychotic-treated schizophrenia patients, combined in the form of an improvement in glycemia [41]. The glycemia improvement must have been also aided by the reduction in consumption of total fats (Table 1) and replacing saturated fatty acid sources with unsaturated fatty acid sources. They affect the structure of cell walls and insulin receptor activity and thus trigger glucose metabolism disorders [42], generating insulin resistance [43]. On the other hand, effects of reduced caffeine uptake (due to undesired interactions with the antipsychotics clozapine, olanzapine, haloperidol, and quetiapine) to the amount contained in 1-2 cups of coffee could hardly be assessed in light of the available literature evidence. Caffeine is known to inhibit A1 adenosine receptor-dependent glucose absorption by muscles and increases insulin resistance associated with increased adrenaline concentration. However, as coffee contains, inter alia, antioxidants and other compounds involved in glucose metabolism, it may counteract the undesirable caffeine effects [44].

The types and sources of carbohydrates used, in combination with five meals served every 2.5-3 hours, acted in favor of maintaining a stable blood glucose level, which not only affected hunger center activity, but enhanced the patients’ sense of wellbeing. A possibility of influencing schizophrenia patients’ hunger- and satiation-controlling centers this way could be important, because such patients usually show an increased appetite, which promotes body weight increase additionally stimulated by antipsychotics [45], with a simultaneous absence of response to increasing concentration of appetite- and energy release-controlling leptin [46]. 

A reduction in the blood glucose concentration has been shown to decrease the secretion of lipopoietic insulin [47], known to stimulate triacylglycerol biosynthesis [48]. The increased concentration of insulin produced by diet composition triggers the pentose cycle, which supplies materials for fatty acid synthesis; it also stimulates fatty acid esterification by facilitating glucose absorption by adipocytes and by activating glycerol-3-phosphate acetyltransferase. 

That such a mechanism was active is suggested by the significant reduction in the triacylglycerol concentration in all the males and in most of the female patients examined. 

Effects of diet modification were visible as a reduction of total cholesterol and LDL cholesterol in most of the female patients and in all the men, the reduction being significant in the latter. In light of the results of the research conducted by Pillinger et al. [49], the meta-analysis showed that male gender is a predictor of biochemical metabolic disorders induced by antipsychotic drugs, and the obtained effect can be considered very promising.

Consumption of saturated lipids has been, for a long time, known to be directly correlated with increased blood lipid metrics [50]. A decrease in total fat consumption, especially sources of saturated fatty acids, not only contribute to normalizing glucose concentration, but also result in a reduction of total cholesterol, due mainly to the reduction of LDL cholesterol concentration. The concentration of HDL cholesterol was not significantly different, but the diet modification resulted in an increase in HDL cholesterol to total cholesterol ratio in over half the female and male patients. The effect observed could have been related to a specific influence of increased consumption of unsaturated fatty acids (contained in olive and rapeseed oils and fatty fish) and higher amounts of vitamin C-containing vegetables. 

Active in reducing the total cholesterol concentration in the patients were numerous other nutrition-related factors, such as a substantial increase in dietary fiber uptake (Table 1). Dietary fiber is known to assist in the removal of bile acids and their salts from the intestine and to direct cholesterol to the bile acid pool; this reduces the amount of bile acids available for incorporation into lipoproteins [51]. Effects of the increased consumption of fiber, its soluble fractions in particular [52], were additionally intensified by the increased consumption of vitamin C (Table 3), assisting in regulating the activity of HMG-CoA reductase, a key cholesterol synthesis pathway enzyme [53]. 

The total cholesterol reduction could have also been assisted by the reduced consumption of animal proteins (Table 1) on the one hand and by the increased intake of folates and vitamin B6 on the other. The vitamins in question are important in protein (particularly methionine) metabolism, and should their uptake be uncorrelated with the dietary protein content, methionine would not be properly metabolized, and homocysteine would be produced. Excessive concentration of homocysteine adversely affects, inter alia, the vascular endothelium and may be involved in an increase in the blood’s total cholesterol concentration [53]. 

The beneficial effect of the diet on cholesterol concentration could also result from the increased consumption of dietary sources of choline, which is involved in the transport and metabolism of lipids and cholesterol.

Individual patient-reported normalization of defecation, including timing and frequency, suggest improvements in gut microbiota and colon function that may have resulted from a reduction in simple sugar and animal protein intake and an increase in probiotic and dietary fiber intake. Research reviews conducted by Majewska et al. [54] and Sonnenburg et al. [55] indicate that the intestinal microbiota is involved in carbohydrate–lipid metabolism and may contribute to weight loss [56,57]. Disorders of the gut–brain axis may also play a role in schizophrenia etiopathogenesis [58].

The direction of metabolic changes in the examined patients was also significantly affected by changes resulting from the increased intake of vitamins, minerals and other biologically active ingredients, including vitamin C with antioxidant activity, vitamin B6 involved in the metabolism of carbohydrates, and vitamin D, the concentration of which is correlated with the concentration of HDL cholesterol [59]. Supplementation with calcitriol used in the subjects could also increase the absorption of calcium, which is involved in lipid metabolism. Dietary calcium deficiency triggers the endogenous synthesis of calcitriol, which increases the uptake of calcium ions by cells, the rate of lipogenesis, and the accumulation of adipose tissue [60]. In addition, the changes in body weight and waist circumference could have been important for the parameters measured by significantly altering cell sensitivity to insulin and glucose absorption by the liver. However, all the cause–effect relationships between diet composition and the changes observed merit a much more voluminous discussion than that which is possible here. 

A weaker effect of diet correction on the parameters examined in female patients is a gender-specific outcome, gender specificity being a stronger recognized risk factor in schizophrenia-related metabolism disorders [61] than the female patients’ readiness to follow the dietary recommendations. It is in women where weight gain induced by antipsychotics may be more frequent [62]. Losing weight and reducing waist circumference were one of the strongest factors motivating adherence to the recommendations (which included, inter alia, giving up purchases of sweets and caffeine-containing sweet beverages, limiting coffee drinking to 1 cup per day, and consumption of the recommended amounts of vegetables and fruits). The authors’ earlier research demonstrates weight loss awareness and the desire to lose weight to be the most important for women, regardless of actual body weight, age, or health state, including mental illness, as shown in this study [63,64,65,66].

The altered dietary habits produced many other beneficial changes in body functions, reported both by the patients and by the NH staff and physicians. The changes were in many cases limited to some patients, but they were always important for the person concerned and improved their quality of life.

Taking into account the type of changes introduced, mainly consisting in changing the sources of nutrients and changes in food preparation technology, it seems that they can be introduced into similar nursing homes, i.e., full-day care and with such a number of residents that allows for personalized care. This is related to the way of introducing changes consisting in acquainting residents with them, obtaining their consent and acceptance, continuous education, but also including in occupational therapy the preparation of dishes that the residents like the most and which do not belong to the group of recommended dishes. In the NH under study, these were potato pancakes, served (after draining the excess fat on tissue paper) once per month, in two versions—sweet and dry.

The limitation of the study was the lack of a control group and the size of the groups, resulting from the residents currently residing in the nursing home.

## 5. Conclusions

The results obtained allow us to conclude that:

1. Schizophrenia patients are capable of adhering, with full acceptance and cooperation, to principles of appropriate nutrition.
2. Nutrition effects are strong enough to produce a significant reduction in blood glucose concentration to the reference level, as observed in all the patients, regardless of the antipsychotic used in schizophrenia treatment.
3. The glucose concentration reduction found enhances blood lipid indicators, but the reduction in triacylglycerols, total cholesterol and LDL cholesterol was significant in male patients only.
4. Nutritional changes were reflected, in overweight and obese women only, in body weight reduction and waist adipose tissue loss.

## Figures and Tables

**Table 1:** The content of energy and basic nutrients in all-day food rations, including disc remnants, of women and men; $\overset{-}{x}$ ± SD, % of the recommended norm.

Component	Women		SS	Men		SS
	before *n* = 360	after *n* = 360		before *n* = 540	after *n* = 540	
Energy (MJ) (%)	12.6 ± 1.14 156 ± 12.7	11.5 ± 0.79 142 ± 11.5	** **	12.6 ± 2.31 128 ± 26.0	11.6 ± 1.69 118 ± 19.9	** **
Total protein (g) (%)	93.2 ± 5.84 128 ± 11.1	93.4 ± 6.0 128 ± 11.4	- -	93.5 ± 13.9 95.5 ± 13.9	105 ± 17.5 108 ± 16.6	** **
Animal protein (g) (%)	50.1 ± 3.07 138 ± 14.1	46.9 ± 4.17 129 ± 14.0	** **	52.9 ± 10.1 179 ± 33.1	49.7 ± 12.4 168 ± 39.8	** **
Fat (g) (%)	104 ± 8.99 162 ± 14.3	83.7 ± 7.91 130 ± 15.8	** **	107 ± 27.3 137 ± 36.6	81.9 ± 11.8 104 ± 16.0	** **
Cholesterol (mg) (%)	350 ± 40.7 117 ± 13.6	307 ± 27.2 102 ± 9.05	** **	354 ± 58.1 118 ± 19.4	307 ± 39.9 102 ± 13.3	** **
Carbohydrates (g) (%)	462 ± 48.8 173 ± 16.3	454 ± 33.6 170 ± 12.0	- -	449 ± 74.1 128 ± 30.2	461 ± 66.7 131 ± 25.4	** **
Fiber (g) (%)	39.5 ± 8.42 157 ± 33.7	53.4 ± 8.07 213 ± 32.2	** **	36.0 ± 7.97 144 ± 31,9	50.9 ± 6.6 203 ± 26.4	** **
Liquids (ml) (%)	2015 ± 270 101 ± 13.5	2597 ± 340 130 ± 17.0	** **	2052 ± 325 82.1 ± 13.0	2603 ± 418 104 ± 16.7	** **

SS: statistical significance. ** statistically significant difference, *p* ≤ 0.01.

**Table 2:** Percentage share of energy from basic nutrients in all-day food rations, including disk remnants of women and men, $\overset{-}{x}$ ± SD.

The Share of Energy from:	Women		SS	Men		SS
	before *n* = 360	after *n* = 360		before *n* = 540	after *n* = 540	
Proteins	12.4 ± 0.66	13.9 ± 0.57	**	12.5 ± 0.69	13.8 ± 0.72	**
Lipids	31.2 ± 1.89	27.3 ± 1.66	**	32.0 ± 3.0	26.7 ± 1.60	**
Carbohydrates	56.4 ± 2.13	58.8 ± 1.65	**	55.5 ± 2.61	59.5 ± 1.94	**
Saccharose	17.8 ± 1.52	9.9 ± 0.69	*	18.9 ± 1.23	9.4 ± 0.58	-

SS: statistical Significance. *, ** statistically significant difference, * *p* ≤ 0.05; ** *p* ≤ 0.01.

**Table 3:** Content of selected vitamins and minerals in all-day food rations, including disk remnants, of women and men; $\overset{-}{x}$ ± SD, % of the recommended norm.

Component	Women		SS	Men		SS
	before *n* = 360	after *n* = 360		before *n* = 540	after *n* = 540	
Folate (µg) (%)	443 ± 35.0 138 ± 10.9	603 ± 52.2 188 ± 16.2	** **	442 ± 57.2 138 ± 17.9	606 ± 55.4 189 ± 17.3	** **
Vitamin B_1_ (mg) (%)	1.62 ± 0.155 180 ± 17.2	1.76 ± 0.137 195 ± 15.2	** **	1.64 ± 0.279 149 ± 25.4	1.80 ± 0.19 163 ± 17.7	** **
Vitamin B_2_ (mg) (%)	1.86 ± 0.177 207 ± 19.7	2.10 ± 0.23 233 ± 25.6	** **	1.85 ± 0.23 168 ± 21.1	2.10 ± 0.39 191 ± 35.9	** **
Vitamin B_6_ (mg) (%)	2.30 ± 0.231 181 ± 23.3	2.71 ± 0.33 213 ± 25.7	** **	2.34 ± 0.38 176 v 28.3	2.76 ± 0.36 211 ± 30.9	** **
Vitamin C (mg) (%)	96.8 ± 43.4 161 ± 72.3	181 ± 28.4 302 ± 47.3	** **	102 ± 77.7 136 ± 104	191 ± 77.3 255 ± 103	** **
Vitamin D ^1^ (µg) (%)	2.10 ± 0.20 21.0 ± 1.98	2.79 ± 0.47 27.9 ± 8.77	** **	2.54 ± 1.62 25.3 ± 16.2	3.07 ± 2.07 30.7 ± 20.7	** **
Calcium (mg) (%)	569 ± 82.7 58.7 ± 9.21	751 ± 150 77.8 ± 18.4	** **	561 ± 122 68.2 ± 17.2	742 ± 206 89.3 ± 28.4	** **
Magnesium (mg) (%)	412 ± 61.2 155 ± 23.1	471 ± 69.8 177 ± 26.3	** **	395 ± 77.8 113 ± 22.0	463 ± 66.8 133 ± 18.8	** **
Iron (mg) (%)	16.9 ± 2.27 272 ± 46.0	18.8 ± 2.25 303 ± 49.7	** **	16.4 ± 2.46 273 ± 41.1	18.6 ± 1.95 311 ± 32.5	** **
Zinc (mg) (%)	15.0 ± 2.49 220 ± 36.6	15.0 ± 2.41 220 ± 35.5	- -	14.4 ± 2.89 211 ± 42.5	14.7 ± 2.38 217 ± 34.9	- -
Cooper (mg) (%)	1.73 ± 0.228 192 ± 25.3	2.12 ± 0.246 235 ± 27.4	** **	1.68 ± 0.275 186 ± 30.6	2.08 ± 0.225 231 ± 25.1	** **

^1^ Vitamin D content was given without taking into account liver oil supplementation. SS: statistical significance. ** statistically significant difference, *p* ≤ 0.01.

**Table 4:** The impact of changes in the diet on the value of the glycemic load, including disc remnants, of women and men; $\overset{-}{x}$ ± SD.

Trait	Women		SS	Men		SS
	before *n* = 360	after *n* = 360		before *n* = 540	after *n* = 540	
Breakfast	60.2 ± 15.9	54.8 ± 7.0	*	64.6 ± 13.3	56.5 ± 6.4	*
Lunch	6.0 ± 8.3	3.0 ± 5.4	-	3.7 ± 7.5	2.4 ± 4.8	-
Dinner	42.9 ± 0.3	43.9 ± 0.05	-	43.6 ± 0.2	43.5 ± 0.01	-
Afternoon snack	0.0 ± 0.0	12.0 ± 0.05	**	0.0 ± 0.0	13.2 ± 0.04	**
Supper	62.3 ± 17.8	58.5 ± 6.6	-	65.6 ± 16.1	59.9 ± 6.1	-
Snacks	76.1 ± 36.9	47.8 ± 20.1	**	84.5 ± 58.0	53.1 ± 33.6	**
Σ of 24 hours	247.3 ± 45.7	220.0 ± 21.9	**	262.1 ± 61.7	228.6 ± 35.2	**

SS: statistical significance. *, ** statistically significant difference, * *p* ≤ 0.05; ** *p* ≤ 0.01.

**Table 5:** Impact of diet correction on selected anthropometric parameters of nutritional status, DPS patients suffering from schizophrenia; $\overset{-}{x}$ ± SD, min–max.

Trait	Women, *n* = 12		SS	Men, *n* = 18		SS
	before	after		before	after	
Body weight (kg)	77.9 ± 18.8 56–118	75.2 ± 17.6 56–112	*	79.1 ± 9.7 64–102	79.3 ± 12.0 62.7–106	-
BMI (kg/m^2^)	30.1 ± 7.8 20.6–51.1	29.1 ± 7.3 19.3–48	*	27.2 ± 3.8 21.5–36.6	27.3 ± 4.4 22.5–38.0	-
WC (cm)	100.6 ± 14.4 78–135	98.9 ± 14.6 71–130.5	-	100.2 ± 8.7 89.0–114	101.0 ± 9.8 90.5–129	-
HC (cm)	108.3 ± 13.3 91–140	104.6 ± 13.2 91–140	*	104.0 ± 6.4 93–118	101.3 ± 7.7 90–117	-
WHR	0.93 ± 0.06 0.79–1.0	0.95 ± 0.08 0.77–1.07	-	0.96 ± 0.06 0.86–1.07	1.00 ± 0.05 0.92–1.11	-
WHtR	0.63 ± 0.10 0.45–0.89	0.62 ± 0.11 0.41–0.86	-	0.59 ± 0.07 0.49–0.72	0.59 ± 0.07 0.51–0.72	-

SS: statistical significance, BMI: Body Mass Index, WC: waist circumference, HC: hip circumference, WHR: waist-to-hip ratio, WHtR: waist-to-height ratio, * statistically significant difference, *p* ≤ 0.05.

**Table 6:** Impact of diet correction on selected parameters of carbohydrate–lipid metabolism, DPS patients suffering from schizophrenia; $\overset{-}{x}$ ± SD, min–max.

Trait	Women, *n* = 12		SS	Men, *n* = 18		SS
	before	after		before	after	
Glucose (mmol/L)	4.96 ± 0.89 3.83–6.89	4.42 ± 0.76 3.56–6.22	*	4.97 ± 1.08 4.11–8.39	4.14 ± 0.61 3.22–5.28	**
TG (mmol/L)	2.05 ± 0,85 0.88–4.15	1.58 ± 0.63 0.82–2.79	-	1.92 ± 1.07 0.67–4.02	1.52 ± 0.75 0.61–3.56	*
TC (mmol/L)	5.43 ± 1.39 3.26–7.59	4.76 ± 0.85 3.41–6.16	-	5.07 ± 1.08 3.16–6.83	4.41 ± 0.91 2.67 –5.62	*
HDL-C (mmol/L)	1.17 ± 0.35 0.72–1.93	1.24 ± 0.33 0.84–1.78	-	1.11 ± 0.39 0.75–1.88	1.09 ± 0.42 0.46–1.84	-
LDL-C (mmol/L)	3.25 ± 1.14 1.58–4.25	2.81 ± 0.79 1.58–4.25	-	3.19 ± 0.98 1.58–4.87	2.71±0.86 1.48–4.17	**
HDL-C/TC	0,229 ± 0,094 0,122–0,455	0,279 ± 0,009 0,137–0,443	-	0,235 ± 0,102 0,124–0,427	0,258 ± 0,107 0,126–0,482	-

SS: statistical significance, TG: triacylglycerols, TC: total cholesterol, HDL-C: high-density lipoprotein cholesterol fraction, LDL: low-density lipoprotein cholesterol fraction, *, ** statistically significant difference, * *p* ≤ 0.05; ** *p* ≤ 0.01.

## Data Availability

Not applicable.
